# Environmental Impact of the Production of Mealworms as a Protein Source for Humans – A Life Cycle Assessment

**DOI:** 10.1371/journal.pone.0051145

**Published:** 2012-12-19

**Authors:** Dennis G. A. B. Oonincx, Imke J. M. de Boer

**Affiliations:** 1 Department of Plant Sciences, Wageningen University, Wageningen, The Netherlands; 2 Animal Department of Animal Sciences, Wageningen University, Wageningen, The Netherlands; Texas A&M University, United States of America

## Abstract

The demand for animal protein is expected to rise by 70–80% between 2012 and 2050, while the current animal production sector already causes major environmental degradation. Edible insects are suggested as a more sustainable source of animal protein. However, few experimental data regarding environmental impact of insect production are available. Therefore, a lifecycle assessment for mealworm production was conducted, in which greenhouse gas production, energy use and land use were quantified and compared to conventional sources of animal protein. Production of one kg of edible protein from milk, chicken, pork or beef result in higher greenhouse gas emissions, require similar amounts of energy and require much more land. This study demonstrates that mealworms should be considered a more sustainable source of edible protein.

## Introduction

The demand for food of animal origin is rising globally and is expected to increase by 70–80% between 2012 and 2050 [1], [2], [3]. Currently, the livestock sector uses about 70% of all agricultural land [2], [4] and is responsible for about 15% of the total emission of anthropogenic greenhouse gas (GHG) [2], [3]. Expansion of agricultural acreage by land clearing is a major source of GHG emissions [2], [5] and one of the largest contributors to global warming [6]. People's choices for certain diets influence GHG emissions and other environmental parameters [6], [7]. A suggested mitigation measure is a shift towards protein from lower impact animal species [1], [4], [8]. Various authors have suggested insects as an environmentally more friendly alternative to conventional livestock [9], [10], [11], [12], [13]. However, little data are available on the environmental impact associated with insect production. Husbandry contributions to GHG emissions is much lower for insects (2–122 g/kg mass gain) than for beef cattle (2850 g/kg mass gain), and in the lower range when compared to pigs (80–1130 g/kg mass gain) [14]. However, this is only a part of the total GHG emissions in animal production chains. To choose among different sources of animal protein, GHG emissions, and other environmental parameters, such as land or fossil energy use, need to be assessed. Life cycle assessment (LCA) is a widely accepted method to quantify these parameters [15] and has been used for various animal products [16]. Within an LCA preselected parameters are quantified along the entire life cycle of a product. For mealworms, for instance, not only direct GHG emissions through respiration, but also GHG emissions related to feed production, and distribution as well as emissions due to the heating of the climate-controlled-rearing facility are quantified and attributed to a product. Although claims that insects are a more sustainable protein source than conventional livestock are widespread, to our knowledge, an LCA of any insect species used as a protein source has never been published. The objective of this paper, therefore, was to quantify the environmental impact attributed to the production of two tenebrionid species, viz. the mealworm (*Tenebrio molitor*) and the super worm (*Zophobas morio*). This impact was then compared to conventional sources of animal protein, such as milk, chicken, pork and beef.

## Materials and Methods

**Figure 1:**
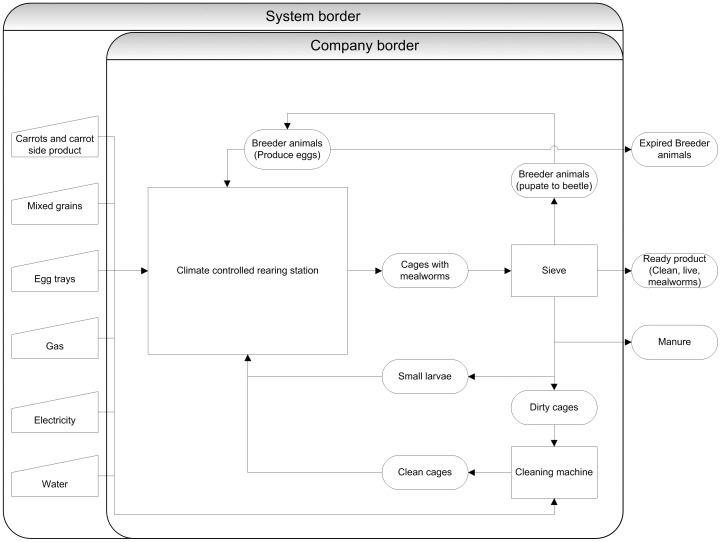
The mealworm production system. Flows entering the company are on the left, centrally the production steps are shown and flows exiting the system are on the right. For flow quantities see Table 1.

Through collaboration with a commercial mealworm producer in The Netherlands (van de Ven Insectenkwekerij, Deurne, The Netherlands), insight in the production process of mealworms was acquired. This farm produces two mealworm species in the same way and at equal quantity (kg/year). Therefore, we conducted a combined LCA for both species. First, the system boundary was defined (Figure 1). A cradle-to-farm-gate approach was chosen, which means that the environmental impact was assessed up to the moment that the fresh product leaves the farm gate.

An LCA relates the environmental impact of a product to a functional unit (FU). Comparing LCA results among animal products demands identical FU's. Mealworms, like other animal products, can nutritionally be seen as a source of protein. Therefore, we defined two FUs in our study: 1) kg of fresh product, and 2) kg of edible protein. To compute the amount of edible protein, we first multiplied the kg of fresh product with the average reported dry matter (DM) content (*T. molitor* 38%; *Z. morio* 43%) and the average percentage of reported crude protein in the dry matter (*T. molitor* 53%; *Z. morio* 45%) [17], [18], [19], [20]. Subsequently, we multiplied with the edible portion, which we consider to be 100% for mealworms since they are consumed by humans as the whole animal. Protein content [16], [21], [22] and edible portion [16], [22] of common production animals vary depending on breed, country of production and other factors. In this study we used the data reported by De Vries & De Boer [16].

We quantified three environmental indicators: 1) global warming potential (GWP), 2) fossil energy use (EU), and, 3) land use (LU). Global warming potential was expressed in CO_2_-equivalents (CO_2_-eq); the sum of CO_2_, CH_4_, and N_2_O emissions. The conversion factor to CO_2_-eq is 1 for CO_2_, 25 for CH_4_ and 298 for N_2_O [23]. Land use was expressed in m^2^ per year, and fossil energy use in mega joules (MJ). When several products stem from one production process, such as grain and straw, it is called a multifunctional production process. Its environmental impact is then allocated to the various outputs. Multifunctional processes included in our system were: (1) production of feed ingredients and their co-products, and (2) production of mealworms and its co-product manure. We allocated the impact of feed production to its outputs based on their relative economic value, whereas the impact of mealworm production was fully allocated to mealworms.

All inputs of the mealworm production system were quantified in this assessment. The production system, including the diet, is identical for both mealworm species (Figure 1). The diet consisted of fresh carrots and a mixed grain feed (i.e. wheat bran, oats, soy, rye and corn supplemented with beer yeast). For industrial competitive protection the exact composition of the diet is not disclosed. The feed conversion ratio (FCR) for concentrates was calculated by dividing the amount of concentrates used by the amount of live mealworms produced. Egg cartons are used to increase the surface area for the adult mealworms. For the environmental impact of the egg cartons, data for recycled cardboard were used. A batch of twenty egg cartons was dried at 70°C until a stable weight was reached and the average dry weight was assumed representative.

In order to create an optimal rearing environment, the climate-controlled-rearing facility is heated and ventilated by the usage of natural gas and electricity from the Dutch power grid. In order to minimize seasonal influences on energy usage, data for a complete year were used.

The quantities of all inputs and the output of the production system were disclosed by the mealworm producing company. Quantitative data for each input regarding GWP, EU, and LU (Table 1) were based on Ecoinvent [24] and new data from the Dutch animal feed industry [25]. This data includes: the production, processing, and transportation of carrots, feed ingredients, and egg trays as well as the production, transportation and use of natural gas, electricity and water. The LU of the farm and the direct GHG emissions from the mealworms were added to this data. Direct GHG emission for *Z. morio* and *T. molitor* were assumed equal and data from Oonincx et al. (2010b), in which the same diet was used, were assumed representative. Finally, the total GWP, EU, and LU were divided by kg of fresh mealworm, or the kg of edible protein produced, resulting in an expression for both FUs.

**Table 1:** Resource use per year and environmental impact.

Resource	Turnover/Year	GWP	EU	LU
		kg CO_2_-eq	MJ	m^2^
Carrots (kg)	260000	0.12	1.38	0.16
Mixed grains (kg)	182000	0.51	4.79	1.39
Egg trays (kg)	262	0.98	13.70	0.10
Gas (MJ)	811200	0.07	1.20	0.00
Electricity (MJ)	187200	0.20	3.17	0.00
Water (M^3^)	211	0.32	5.55	0.04
Animal (kg)	83200	0.01	0.00	0.00
Farm	1	0	0.00	588

Global warming potential (GWP), energy use (EU) and land use (LU) are expressed per unit of input based on economic allocation.

## Results

The Feed Conversion Ratio (FCR) for concentrates was 2.2 kg/kg of live weight in this study. The absolute and relative GWP, EU, and LU for the production of one kilogram of fresh mealworms based on economic allocation are provided in Table 2. The GWP of one kg of fresh mealworms was 2.7 kg of CO_2_-eq, of which 42% results from the production and transport of feed grains, 14% from the production and transport of carrots, 26% from gas used for heating, and 17% from the use of electricity. The EU of one kg of fresh mealworms was 34 MJ, of which 31% results from the emission of production and transport of feed grains, 13% from the production and transport of carrots, 35% from gas used for heating, and 21% from the use of electricity. The LU of one kg of fresh mealworms was 3.6 m^2^ per year, of which 85% was required to cultivate feed grains, and 14% to produce carrots. When expressed per kg of edible protein from mealworms, the GWP was 14 kg of CO_2_-eq, the EU was 173 MJ and the LU was 18 m^2^. The relative contributions remain the same.

**Table 2:** Environmental impact of inputs in a mealworm production system.

	GWP (kg CO_2_-eq)		EU (MJ)		LU (m^2^)	
Carrots (kg)	0.38	14.27%	4.31	12.80%	0.51	14.39%
Mixed grains (kg)	1.11	41.98%	10.47	31.09%	3.03	85.14%
Gas (MJ)	0.70	26.26%	11.71	34.77%	0.00	0.02%
Egg trays (kg)	0.00	0.12%	0.04	0.13%	0.00	0.01%
Electricity (MJ)	0.45	17.06%	7.13	21.17%	0.01	0.24%
Water (M^3^)	0.00	0.03%	0.01	0.04%	0.00	0.00%
Animal (kg)	0.01	0.29%	0.00	0.00%	0.00	0.00%
Farm	0.00	0.00%	0.00	0.00%	0.01	0.20%
Total	2.65	100.00%	33.68	100.00%	3.56	100.00%

Absolute and relative contribution global warming potential (GWP), energy use (EU) and land use (LU) for the production of one kg of fresh mealworms based on economic allocation.

## Discussion

Both mass and economic allocation can be used when describing the environmental impact of a product. Since the latter is more commonly used, values in this discussion are based on economic allocation allowing comparison with other food sources of animal origin.

Differences in environmental impact of products from pork, chicken, and beef are caused by three main factors; 1) enteric CH_4_ production, 2) reproduction rate and 3) feed conversion efficiency [16]. Based on these three factors one would expect a lower environmental impact from mealworm production. Firstly, mealworms do not produce CH_4_ [14], secondly they have a high reproduction rate; one female of *T. molitor* produces 160 eggs in her life (3 months) and a *Z. morio* female produces1500 eggs in her life (1 year). Furthermore, the maturation period is short; *T. molitor* reaches adulthood in 10 weeks and *Z. morio* in 3.5 months [27]. Thirdly, feed conversion efficiency, depends amongst other things, on the diet provided. The feed conversion ratio (FCR) for concentrates (kg/kg of fresh weight) for the mealworms in this study (2.2) was similar to values reported for chicken (2.3) but lower than for pigs (4.0) and beef cattle (2.7–8.8) [28]. The large spread reported for beef cattle is due to variation in the proportion of concentrates, relative to roughage (for instance grass), used in the diet.

**Figure 2:**
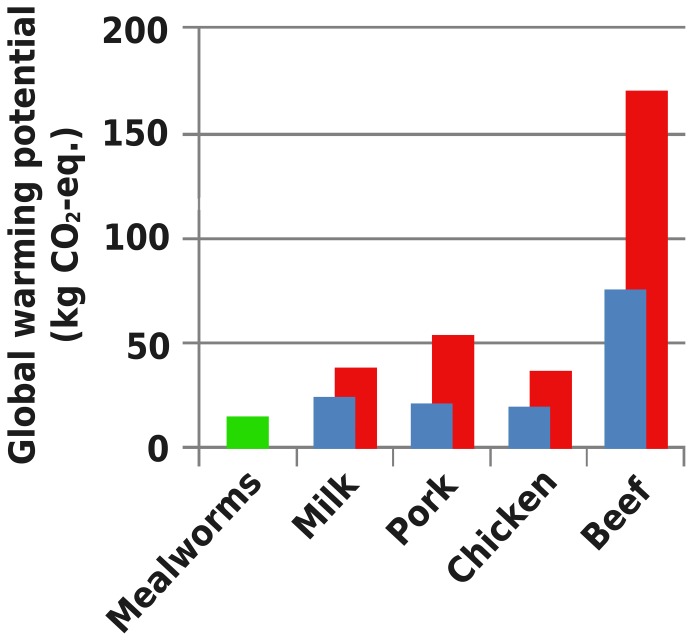
Environmental impact of mealworms compared to other animal products. Global warming potential due to the production of one kg of edible protein. Results from this study depicted in green. Minimum (blue) and maximum (red) literature data is adapted from de Vries & de Boer (2010).

**Figure 3:**
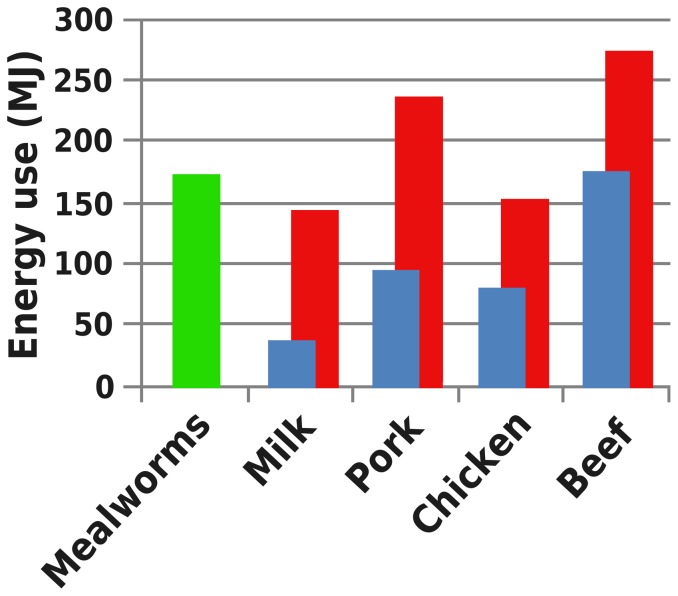
Environmental impact of mealworms compared to other animal products. Energy use due to the production of one kg of edible protein. Results from this study depicted in green. Minimum (blue) and maximum (red) literature data is adapted from de Vries & de Boer (2010).

**Figure 4:**
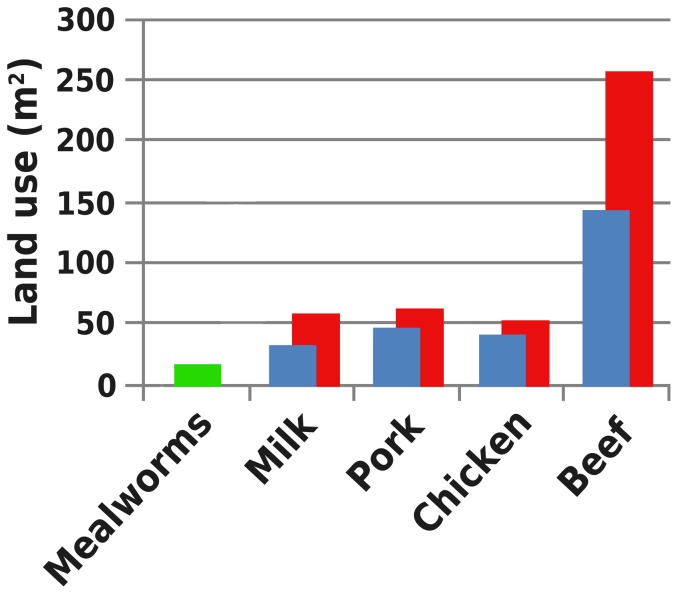
Environmental impact of mealworms compared to other animal products. Land use due to the production of one kg of edible protein. Results from this study depicted in green. Minimum (blue) and maximum (red) literature data is adapted from de Vries & de Boer (2010).

We assessed three indicators to provide insight in the sustainability of mealworm production and compared these to literature values (Figure 2, 3 and 4). The GWP of mealworms per kg of edible protein is low compared to milk (1.77–2.80× as high), chicken (1.32–2.67× as high), pork (1.51–3.87× as high) or beef (5.52–12.51× as high) [16]. The EU of mealworm production per kg of edible protein is higher than for milk (21–83% of the value for mealworm) or chicken (46–88% of the value for mealworm), similar to pork (55–137% of the value for mealworm) and lower than for beef (1.02–1.58× as high). Mealworms, being poikilothermic, depend on suitable ambient temperatures for growth and development. When ambient temperatures are low, heating is required, increasing energy use. Mitigation measures are being investigated: larger larvae in this system produce a surplus of metabolic heat, which could be used to heat the heat-demanding small larvae. The LU of the described production system was very low compared to milk (1.81–3.23× as high), chicken (2.30–2.85× as high), pork (2.57–3.49× as high higher) and beef (7.89–14.12× as high).

Over the last two decades productivity of chickens and pigs has increased annually by 2.3%, due to the application of science and new technologies [29]. Further improvement of the mealworm production system by, for instance, automation, feed optimization or genetic strain selection is expected to increase productivity and decrease the environmental impact. Since these aspects are currently underdeveloped, the potential rate of productivity improvement is expected to be higher for mealworms compared to the more common production animals.

Two further aspects can influence the environmental impact of mealworm production: off-farm differences in storage and processing, and the economic value of by-products. Processing and storage has a large effect on the environmental impact of a food product: emissions during slaughtering, transportation and storage of pork and chicken contribute 17–25% to the total GWP [7], [16]. For mealworms, used for human consumption, there is currently no standard method for processing and storing. If the environmental impact due to processing and storage is similar to that of other animal derived food products, the results of this study are also representative beyond the farm gate.

Besides food, there are also non-food by-products from common production animals, such as leather, blood meal or feathers. The relative value of these products is not taken into account for the expression of the selected indicators, expressed per kg of edible protein. A decrease of the environmental impact due to the economic value of these by-products would be relatively small (3% for pork, 5% for chicken, and 3–5% for beef [26]).

We consider the low LU of mealworms to be particularly important; effects of GHG emissions can be countered by carbon fixation [30], and forest regrowth and afforestation [5], [31], depletion of fossil fuels can be countered by usage of alternative sources of energy [32], [33], but land availability is fixed and limited. Expansion of agricultural land is a major source of GHG production especially in tropical regions [4], [5], [6]. Slowing down the expansion of agricultural land is a critical step towards sustainable agriculture [4]. The increasing world population will therefore need to be fed using the same area of land that is available now [6]. Mealworms require only 43% of the amount of land used for the production of one kg of edible animal protein as milk, and only 10% of the land used for production of beef.

### Conclusions

The EU of mealworm production is higher than for milk or chicken and similar to pork and beef. However, mealworms, when considered as a human protein source, produce much less GHG's and require much less land, than chickens, pigs and cattle. With land availability being the most stringent limitation in sustainably feeding the world's population, this study clearly shows that mealworm should be considered as a more sustainable alternative to milk, chicken, pork and beef.
